# Role of infiltrated leucocytes in tumour growth and spread

**DOI:** 10.1038/sj.bjc.6601705

**Published:** 2004-03-30

**Authors:** E Y Lin, J W Pollard

**Affiliations:** 1 Departments of Developmental and Molecular Biology and Obstetrics and Gynecology and Women's Health, Center for the Study of Reproductive Biology and Women's Health and Albert Einstein Cancer Center, Albert Einstein College of Medicine, 1300 Morris Park Ave., Bronx, NY 10461, USA

**Keywords:** Leucocyte, tumour, progression, metastasis, angiogenesis

## Abstract

Leucocytes are a major component of the tumour microenvironment. Recent studies have indicated that the infiltration and activity of these host cells are regulated by the tumour to promote its survival and progression. Through the production of an array of growth factors, proteases and angiogenic mediators, leucocytes in the tumour microenvironment promote tumour growth, angiogenesis and metastasis.

## TUMOUR-ASSOCIATED LEUCOCYTES

The infiltration of leucocytes into solid tumours was remarked upon more than 100 years ago when it was suggested that they had a causal role in carcinogenesis. These infiltrates are now known to contain myeloid cells (neutrophils, dendritic cells, macrophages, eosinophils and mast cells) as well as lymphocytes. However, controversy remains over the relationship between these host cells and tumour progression. In the past, their presence has been construed as evidence for a host response against the growing tumour. This is because such immune functions were often observed in transplantable tumour models that by their nature represented a transplant that could elicit immune rejection despite being placed in immunocompromised hosts. However, it is becoming clear that tumours growing naturally are largely recognized as self and lack strong foreign antigens. Instead, they appear to have been selected to manipulate the host immune system to prevent rejection (Dunn *et al*, 2002) and use this system to facilitate their own growth and spread (Khong and Restifo, 2002). This lack of immune response has become particularly evident with the study of tumours induced by the restricted expression of oncogenes in transgenic mice where it has been established that tumour-associated leucocytes are often active participants in the neoplastic process. In addition, there is a growing body of clinical data on a wide range of solid tumour types that has correlated a high density of leucocytic infiltration with poor outcome (Coussens and Werb, 2001, 2002). Furthermore, it has be recognised that cells containing DNA alterations caused by viral or chemical carcinogens do not progress to become cancerous until they are exposed to a second type of stimulus that often includes chronic irritants or inflammatory agents (Coussens and Werb, 2002). For example, an inflammatory response is required to induce cancers in chickens infected with the potent oncogenic Rous Sarcoma virus despite it carrying the v-*src* oncogene that alone is competent to transform fibroblasts in culture (Sieweke *et al*, 1989). This view of the role of leucocytes in facilitating cancer progression has been further enhanced by the realisation that many cancers are caused or promoted by infectious or other agents that induce chronic inflammation (Coussens and Werb, 2002).

Under normal physiological circumstances, leucocytes are recruited in response to wounding, inflammatory or pathogenic stimuli. They are attracted by the local synthesis of chemokines (chemoattractive cytokines), cytokines and growth factors as well as products of tissue breakdown. These are all part of a signalling system that involves recognition of the pathological state, organisation of an appropriate cellular response and suppression of this response once the situation is resolved. During cutaneous wound healing, these processes require epithelial cell proliferation and migration, angiogenesis and tissue remodelling (Nathan, 2002). In tumours, it is thought that similar chemoattractive factors are also responsible for the recruitment of leucocytes and that these cells play roles comparable to those observed during wound healing. However, because of the accumulation of intrinsic mutations the epithelial cells have lost positional identity and consequently do not stop growing and migrating on cue. Instead, they send out continuous signals that recruit leucocytes to continue to support the tumor's development. This concept has led to the rubric that tumours are ‘wounds that never heal’ (Balkwill and Mantovani, 2001).

Several steps are crucial for a tumour to become metastatic. Tumour cells need to be able to break out of their confining basement membranes in order to enter the extracellular matrix and circulation. These processes require the proteolytic breakdown of basement membranes, changes in epithelial cell adhesion, migration and the suppression of ankoisis. They are matched in the surrounding stroma with angiogenesis as well as the frequent recruitment of leucocytes. Angiogenesis, known to be the crucial process for tumour progression by providing oxygen and nutrients and removal of waste products, as well as, providing an expanding endothelial surface for the tumour cells to enter the circulation, also involves degradation of basement membranes followed by migration of endothelial cells into the tumour stroma (Folkman, 2002). Recent studies have shown that the tumour-associated leucocytes produce factors that promote all these steps associated with malignancy within tumours (Ribatti *et al*, 2001). This review will focus on the evidence that members of the myeloid lineage, particularly macrophages, neutrophils and mast cells, can facilitate tumour progression.

### Macrophages

Macrophages derived from circulating monocytes represent a major component of the infiltrated leucocytic population in the tumour microenvironment. These cells have a wide range of functions in immunity, during development and in tissue repair. They can adopt a particular phenotype according to the demand and produce many factors ranging from chemokines, cytokines, and proteases, to angiogenic and growth factors. They therefore appear to be the ‘jack-of all trades’ of the myeloid lineage. Of all the cells of the myeloid lineage, the evidence is strongest in support of a positive impact of macrophages on tumour progression. For example, in greater than 80% of clinical studies, an increase in tumour-associated macrophages (TAMs) density is correlated with poor prognosis, with less than 10% of studies showing the converse (Bingle *et al*, 2002). Similarly, overexpression of macrophage chemoattractants within tumours has also been shown to correlate with poor prognosis (Leek and Harris, 2002). One such example is colony stimulating factor-1 (CSF-1 or-macrophage CSF), a macrophage growth factor as well as a potent macrophage chemoattractant (Lin *et al*, 2002). Overexpression of CSF-1 correlates with poor prognosis in human breast, ovarian endometrial and prostatic carcinomas (Kacinski, 1997). In breast cancers, this overexpression correlates with a strong leucocytic infiltration in over 95% of cases (Scholl *et al*, 1994). Similarly, the CC chemokine ligand 2 CCL2/MCP-1 (MCP=monocyte chemoattractant protein 1) has been identified as a major chemokine for macrophages recruitment in several human tumours, including the bladder (Amann *et al*, 1998), cervix (Riethdorf *et al*, 1996), ovary (Negus *et al*, 1995), lung (Arenberg *et al*, 2000) and breast (Valkovic *et al*, 1998; Ueno *et al*, 2000). The level of CCL2/MCP-1 expression is correlated with the increased infiltration of macrophage (Ueno *et al*, 2000) and the grade of tumour (Amann *et al*, 1998; Valkovic *et al*, 1998). Although both CSF-1 and CCL-2 can be targeted to the tumour cells themselves, the strong correlation of overexpression of these macrophage chemoattractants with macrophage recruitment and poor prognosis suggests that TAMs can play a major role in the progression of tumours to metastasis.

**Figure 1:**
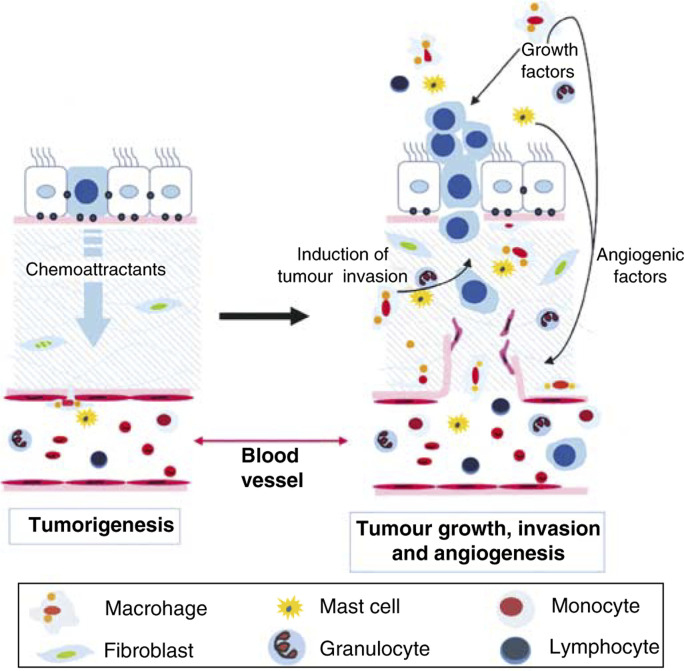
Leucocytic infiltration promotes tumour progression to malignancy. In a manner similar to wounded tissues, solid tumours induce a local ‘inflammatory response’ by attracting leucocytes into its microenvironment. Such an infiltration consists of multiple cell types of which cells of the myeloid lineage are the major component. Leucocytes in such ‘inflammatory sites’ produce an array of growth and angiogenic factors, proteases and mutagenic factors that promote tumour growth, invasion and angiogenesis. However, different from its physiological counterpart whose inflammation ceases when the wound has healed, tumour-induced inflammation persists and eventually leads to tumour progression and metastasis.

Several experiments have supported the role of macrophages in tumour progression. We have observed that in a Polyoma Middle T antigen-induced mouse model of breast cancer (PyMT), an increase of macrophage infiltration at the primary tumour site occurred immediately before the onset of malignant transition (Figure 1

) (Lin *et al*, 2001). Using genetic approaches, we demonstrated that depletion of CSF-1 in this model markedly decreased the infiltration of macrophages at the tumor site and this correlated with a significant delay of tumour progression to metastasis. In contrast, overexpression of CSF-1 in the tumour dramatically increased the macrophage density in the primary tumour and this was correlated with an accelerated malignant switch (Lin *et al*, 2001). Similarly, removal of CSF-1 from transplanted tumours also resulted in an impairment of growth with extensive necrosis and poor vascularisation, phenotypes that could be reversed by treatment of the mice with CSF-1 (Nowicki *et al*, 1996). These studies have provided strong evidence that TAMs promote the tumour progression to malignancy. This conclusion was enhanced by the observations that treatment of mice that had been xenotransplanted with either a human colonic or embryonic tumour with antisense oligonucleotides directed against mouse CSF-1 reduced tumour growth and prolonged survival. This was associated with a reduced serum concentration of CSF-1 and a decreased TAM density. Since mouse CSF-1 does not stimulate the human receptor, these data argue for the effects of the CSF-1 antisense molecules to be mediated through the reduction in TAMs (Aharinejad *et al* 2002).

The evidence from both clinical and experimental studies supports the view that, in most cases, TAMs facilitate tumour progression and metastasis. The mechanism(s) macrophages used to promote tumour progression are still unknown; however, it has been proposed that macrophages may promote tumour growth and angiogenesis through the production of growth factors and angiogenic inducers such as Epidermal Growth Factor (EGF), vascular endothelial growth factor (VEGF), tumour necrosis factor (TNF*α*) and Thymidine Phosphorylase (TP) (Xiong *et al*, 1998; Leek and Harris, 2002). Macrophages also indirectly enhance blood vessel formation by possessing a procoagulant activity through fibrin deposition (Mantovani *et al*, 1992). In addition, many macrophages produced factors, proteases and protease activators such as transforming growth factor-*β* (TGF*β*), platelet-derived growth factor, interleukin-6 (IL-6), urokinase plasminogen activator and Tissue-type Plasminogen Activator (t-PA) that may cause degradation of extracellular matrix to facilitate the tumour cell invasion and migration and induce angiogenesis (Egami *et al*, 2003; Eubank *et al*, 2003; Hildenbrand *et al*, 1995; Klimetzek and Sorg, 1977). Moreover, TAMs contribute greatly to the growth of the tumour by producing proangiogenic and tumour-stimulating chemokines such as CCR2 ligands (Vicari and Caux, 2002).

Macrophages can display tumour cytoxicity and can potentially present tumour antigens to induce specific immune reaction against tumours. However, these cells are believed to have primarily a protumour function since both tumours and TAMs produce potent immunomodulating agents that suppress macrophage tumoricidal activity. Such tumour-produced molecules, including IL-4, IL-6, IL-10, CSF-1, TGF*β* and prostaglandin E2 (PGE2), and TAM-produced factors such as IL-10 and PGE2, contribute to the general immunosuppression of the host as well as the antitumour activity of macrophages (Elgert *et al*, 1998; Mytar *et al*, 2003). Although infiltration of macrophages is usually correlated with poor outcome (Hamada *et al*, 2002), recent studies have also shown that infiltrated macrophages may have an anti tumour action in colorectal cancer (Nakayama *et al*, 2002; Noguchi *et al*, 2003). These observations indicate that the microenvironment of different type of tumours might alter the activities of infiltrated leucocytes from tumour promotion to tumour rejection. This has led to the idea of tumour-educated macrophages whose functions are modified by the local cytokine/chemokine environment (Pollard, 2004). In most cases, this enhances tumour development and directs the local immune system away for an antitumour response (Mantovani *et al*, 2002).

Dendritic cells (DCs) also play an important role in both the activation of antigen-specific immunity and the maintenance of tolerance, providing a link between innate and adaptive immunity. Several clinical studies have reported the presence of DC within human tumours such as the stomach, colon, prostate, kidney, thyroid, breast and melanoma (Tsujitani *et al*, 1990; Enk *et al*, 1997; Troy *et al*, 1998; Bell *et al*, 1999; Lespagnard *et al*, 1999; Schwaab *et al*, 1999, 2001; Scarpino *et al*, 2000). However, the effect of such an infiltration in tumour progression is still not clear. Some of these studies have shown that the infiltration of DC was associated with enhanced patient survival (lung), whereas others showed that DC present in tumour either were minimally activated (Tsujitani *et al*, 1990; Troy *et al*, 1998), had no correlation with metastasis-free or overall survival of the patients (Lespagnard *et al*, 1999) or were converted to ‘silencers’ of antitumour immune responses by tumour-produced factors (Enk *et al*, 1997). In addition, studies have reported that patients with a variety of cancers have impaired function of DCs, indicating a systemic effect of the tumours on DCs (Almand *et al*, 2000). Moreover, recent studies have suggested that, instead of initiating immune responses against tumours, DC in the tumour microenvironment may have the ability to turn off the responding T cells and induce tolerance (Hackstein *et al*, 2001; Vicari and Caux, 2002). The relationship between host DC, lymphocytes and tumour in the process of ‘tumour escape’ from the host immune system has been reviewed in detail recently and will not be discussed here (Khong and Restifo, 2002; Hanahan *et al*, 2003). Nevertheless, the evidence on balance suggests that tumours promote the suppression of these potentially damaging cells while enhancing the trophic nature of macrophages. The hope, however, is that therapeutic modulation of this environment locally could result in TAMs that are tumoricidal and that together with properly matured DCs would present antigens to infiltrating T cells with the consequent rejection of the tumour (Dranoff, 2004).

### Mast cells

An infiltration of mast cells has been found in a variety of human cancers, including non-small-cell lung cancer (Shijubo *et al*, 2003), breast cancer (Kankkunen *et al*, 1997), colorectal cancer (Lachter *et al*, 1995), basal cell carcinoma (Yamamoto *et al*, 1997) and pulmonary adenocarcinoma (Imada *et al*, 2000). The accumulation of mast cells has been associated with enhanced growth and invasion of several human cancers (Ribatti *et al*, 2001). However, there are other studies in colorectal cancer where their presence is indicative of improved prognosis (Nielsen *et al*, 1999). Initial studies using animal models have shown that increasing mast cell density in tumour promoted tumour growth (Roche, 1985), whereas reducing their number inhibited tumour growth and angiogenesis (Starkey *et al*, 1988). The role of inflammatory mast cells in tumour progression of squamous epithelial carcinogenesis was also illustrated recently (Coussens *et al*, 1999). Furthermore, Schwann cell tumours caused by inherited mutations in the NF1 gene do not form in mouse models unless the surrounding stromal cells are at least heterozygous for the mutation. These tumours are highly populated with mast cells and it seems likely that a haploinsufficiency of NFI in these cells is the cause of the tumour formation (Zhu *et al*, 2002).

The best known role that mast cells plays in tumour progression is their ability to induce tumour angiogenesis (Hiromatsu and Toda, 2003). Activated mast cells produce a variety of angiogenic growth factors, including VEGF, basic fibroblast growth factor, IL-8 and TNF*α* (Meininger and Zetter, 1992; Qu *et al*, 1995; Hiromatsu and Toda, 2003). In addition, they can produce specific angiogenic mediators including histamine and heparin, which can stimulate endothelial cell proliferation and may contribute to the hyperpermeable nature of newly formed microvessels during pathological angiogenesis (Ribatti *et al*, 2001), and a variety of proteases, particularly MMP9, which are involved in angiogenesis. How tumour cells regulate the infiltration and activation of mast cells is still not fully understood. However, several types of tumours produce stem cell factor that may have functions in mast cell migration, proliferation and activation (Turner *et al*, 1992). In addition to the promotion of angiogenesis, the activated mast cells are a rich source of cytokines and chemokines such as IL-1, IL-3, IL-4, IL-8, granulocyte–macrophage colony-stimulating factor, TNF*α*, interferon-*γ* (IFN*γ*), CCL-2, Macrophage Inflammatory Protein MIP-1*α* and *β*, many of which can contribute to the tumour microenvironment by enhancing tumour cell growth and invasion either directly or through intermediaries such as macrophages (Burd *et al*, 1989; Selvan *et al*, 1994).

### Neutrophils

The role of neutrophils in tumour progression is still controversial. During immune responses, they are among the first cells to arrive at sites of infection where they are highly bactericidal. They are also involved in cell killing during graft rejection and thus they might be considered as potential antitumour cells. However, clinical studies have been contradictory. The presence of increased numbers of tumour-infiltrating neutrophils was linked to poorer outcome in patients with adenocarcinoma of the bronchioloalveolar carcinoma subtype (Bellocq *et al*, 1998), whereas studies of gastric carcinoma suggested that neutrophil infiltration correlated with good prognosis (Caruso *et al*, 2002). It has been reported that tumours prolong alveolar neutrophil survival through the production of soluble factors (Wislez *et al*, 2001). Using transplantable tumour models, studies have shown that tumour-associated neutrophils were involved in tumour angiogenesis by the production of proangiogenic factors such as VEGF and IL-8 (Schaider *et al*, 2003), proteases such as matrix metalloproteinases (Shamamian *et al*, 2001) and elastases (Iwatsuki *et al*, 2000; Scapini *et al*, 2002). In addition, studies using animal models have also shown that neutrophils may contribute to genetic instability in tumours (Haqqani *et al*, 2000).

Furthermore, neutrophil-recruiting cytokines such as GRO (IL-8 homologues) may also directly stimulate tumour proliferation in melanoma (Haghnegahdar *et al*, 2000). Taken together, an environment that recruits neutrophils might enhance angiogenesis, promote tumour invasion and stimulate growth.

## CONCLUSION

Solid tumours are not just composed of malignant cells, but are complex microcosms of many cell types including a wide range of haematopoietic cells. The evidence described above suggests that cells of the myeloid lineages, particularly macrophages, mast cells and neutrophils, on balance play an active role in enhancing tumour progression and metastatic capacity. This is through their ability to promote angiogenesis and tissue remodelling as well as direct effects on epithelial cell viability, growth and migration. In wound healing or in response to an inflammatory stimulus, a similar panoply of cells is recruited. Sentinel cells, particularly macrophages and mast cells that send out chemotactic signals that in the first wave, bringing in neutrophils and monocytes, initiate this recruitment. These not only eliminate pathogens but also effect tissue repair, a process that involves angiogenesis and an induction of vascular permeability, tissue remodelling and the migration and proliferation of epithelial cells. These events are coordinated by a sophisticated and as yet, not understood language of soluble mediators involving cytokines, chemokines and growth factors.

Similarly, during development, myeloid cells, particularly macrophages, play an important role in tissue formation and their absence often results in attenuated poorly formed structures in tissues as wide ranging as bone, skin and mammary gland (Pollard, 2004). It would seem highly likely that tumours send out signals similar to those found in normal physiology to recruit myeloid cells and instruct them to perform similar tasks in tissue remodelling. However, unlike their normal counterparts, the epithelial tumour cells do not stop growing in response to positional cues and continuously send out signals to demand help from the invading myeloid cells.

The tumour microenvironment also educates those invading cells to promote epithelial growth, viability, motility and invasion. Thus, it is noticeable in many tumour types that there are dense lymphocytic infiltration sites adjacent to areas of basement membrane breakdown and tumour invasion (Figure 1). In our studies of the PyMT oncoprotein-induced mammary cancers, these sites marked the transition from nonmalignant to malignant tumours, suggesting that they had a causal role in this process. Furthermore, it appears in this model that macrophages are the sentinel cells that recruit other myeloid cells. This was confirmed by the ablation of macrophages that stopped the infiltration sites from occurring and which resulted in an inhibition of metastatic capacity (Lin *et al*, 2001, 2003). The challenge, therefore, will be to define whether there are unique phenotypes of these tumour-associated myeloid cells that can distinguish them from those involved in immune or tissue repair responses that could be a target for therapeutic agents. In addition, it will be important to understand the cytokine network in the tumour microenvironment that promotes tumour progression so that it can be tilted away from eliciting tropic activities to one that enhances the detection of the tumours as an aberrant state with the resultant suppression of its development and perhaps immunological rejection of the tumour.

### Note added in proof

Ablation of macrophage recruitment in transplantable breast cancers by a chemokine receptor antagonist significantly inhibited tumour development (Robinson *et al*, 2003). Similarly, inhibition of MMP9 production in tumour-associated macrophages by a hypomorphic Ets-2 mutation also inhibited tumour development in the PyMT mouse model of breast cancer (Man *et al*, 2003). These data confirms the involvement of macrophages in tumour development in mouse models.
